# Views of healthcare professionals on gender roles: A qualitative study

**DOI:** 10.1016/j.heliyon.2023.e18576

**Published:** 2023-07-22

**Authors:** Zeynep Dilşah Karaçam Yilmaz, Tülay Yilmaz, Eda Tokman

**Affiliations:** a Istanbul University-Cerrahpasa, Institute of Graduate Studies, Midwifery, Istanbul, Turkey; b Istanbul University-Cerrahpasa, Department of Midwife, Turkey; c Marmara University, Department of Midwife, Turkey

**Keywords:** Gender role, Medical staff, Midwifery, Quality of health care, Qualitative research

## Abstract

**Problem:**

Gender equality is one of the most important determinants of health. Discriminatory interventions, values, beliefs, and prejudices of healthcare professionals during their service affect health adversely.

**Background:**

There is not adequate information about the views of healthcare professionals on gender roles.

**Aim:**

This study was conducted to understand and define the views of healthcare professionals involved in the health system on gender roles.

**Methods:**

This study was performed using the “phenomenological method,” one of the qualitative research methods. A total of 28 healthcare professionals - 10 midwives, 12 nurses, and 6 *doctors* - were included in the research. Individual in-depth interviews were held with the healthcare professionals included in the study.

**Findings:**

As a result of the descriptive analysis performed after the individual interviews about the views of healthcare professionals on gender roles, 6 main themes were determined. These themes are (1) the neglect of women by society, (2) using force on women, (3) seeing women as sexual objects, (4) having a say in society, (5) glorified masculinity and femininity roles, and (6) gender equality.

**Discussion:**

In this study, healthcare professionals expressed that women were neglected by societies since gender roles were determined by patriarchal societies. This situation adversely influences the health of individuals. Importance should be attached to equality between women and men to improve health. Healthcare professionals who support equality will improve the quality of care.

**Conclusion:**

Healthcare professionals should provide equitable services without discrimination. This equitable care they provide will positively affect the health of individuals.

## 1 Introduction

Biological sex is the term attributed to an individual's reproductive organs and carrying XX or XY chromosomes. Gender includes changes resulting from the psychological, behavioral, and social consequences of the gender perceived by the individual. Unlike sex, which is related to biological attributes, gender has a multifaceted and variable nature [1].

The concept of gender is defined as roles and norms constructed on a social basis, attributed to individuals by their female, male, or diverse identities, as well as society, or applied to individuals based on the gender experienced by the individual [2]. Moreover, it refers to the meanings and expectations attributed by society and culture. Gender differs according to cultural values, time, and place [3].

While defining gender equality, the UN Women states the equal rights, responsibilities, and opportunities of women and men, girls and boys. According to them, equality does not mean that men and women are the same. Equality does not mean that the rights, responsibilities, and opportunities of men and women depend on their birth as men or women. Gender equality means recognizing the diversity of women's and men's groups and paying attention to the interests, needs, and priorities of both women and men. Equality between women and men is considered both a human rights issue and a prerequisite for sustainable human-centered development [4].

In the society we live, gender inequality can be seen in many areas. One of the most evident reflections of gender inequality is in the field of health [5]. Restrictive gender norms and gender inequalities are repeated in health systems, resulting in gender inequality in health [6]. This situation substantially affects the physical, social, psychological, and reproductive health of women [2,5,7]. Gender equality, which has been a guide in feminist literature for a long time, is an approach that should be taken into account in practices aimed at improving health [8].

Possible discriminatory practices, values, beliefs, and prejudices of healthcare professionals in the face of injuries, disabilities, and diseases will affect health negatively [9]. Therefore, healthcare professionals should consider gender equality in all their practices while delivering services [7,10]. Healthcare professionals should know the cultural characteristics of the society they will serve in and be aware of the potential inequalities and the social roles and responsibilities required to increase the status of women in society. They should inform the people in their vicinity about gender equality and follow, learn, and teach gender-sensitive developments. Most importantly, they should deliver services in an egalitarian manner, without discrimination, being aware that they are the service provider themselves [7,9,11,12]. Moreover, when gender equality is ensured by healthcare professionals, the quality of care will improve, and there will be better health outcomes. It will not be possible to fulfill the Sustainable Development Goals within the framework of universal health without addressing the role of restrictive gender norms and gender inequalities in the health system [9,13].

This study was conducted to reveal the views of healthcare professionals involved in the health system on gender roles.

## 2 Methods

### 2.1 Design of the study

This study was carried out using a phenomenological study design, a qualitative research method. Qualitative studies are the review and interpretation of observations in a non-numerical way to discover the meaning and type of relationships. The purpose of qualitative studies is to acquire an understanding of how people make sense of their lives, outline the interpretation process, and express how people interpret their experiences [14]. In the research, the Standards for Reporting Qualitative Research (SRQR) criteria proposed for qualitative studies were followed [15].

### 2.2 Population and sample of the study

The study population is comprised of healthcare professionals (doctors, midwives, and nurses) working in an institution delivering health services within the borders of Turkey. The study sample was determined by snowball sampling, one of the purposive sampling methods, and a total of 28 participants - 10 midwives, 12 nurses, and 6 doctors - who met the inclusion criteria were included in the study.

### 2.3 Interviews and data collection

The data were collected between July 2022 and November 2022 using a personal information form and a semi-structured interview form created to review the views of healthcare professionals on gender roles. The interview form consists of 9 questions about the views of healthcare professionals on gender roles.

### 2.4 Data collection methods

The data were collected using the face-to-face, in-depth interview technique. Prior to the interview, the participants were informed about the research. Since the views of people would be discussed, the interviews were held online through the Zoom program at a time that was suitable for the participant.

### 2.5 Analysis

In this study, descriptive statistics were used in the analysis of demographic data, and “descriptive analysis” was used to analyze qualitative data. The data obtained from the interviews were first written down one by one by the authors who collected the data (ZDKY, ET), and then the analysis (ZDKY, TY) was carried out. While performing thematic coding, MaxQDA18 software (Verbi Software, 2018), a qualitative data analysis program, was used.

#### 2.5.1 Validity and reliability

To provide the external validity of the study, examples taken from the answers of healthcare professionals to the interview questions were presented, and thus, the external validity of the study was provided through “direct quotations."

## 3 Findings

In this section of the study, sociodemographic characteristics of healthcare professionals and their views on gender roles are stated.

### 3.1 Participant characteristics

When the sociodemographic characteristics of the healthcare professionals participating in the study were reviewed, it was revealed that their mean age was 30.5 ± 6.73 years, 10.7% (n = 3) were male, 89.3% (n = 25) were female, 32.14% (n = 9) had postgraduate education, and 35.71% (n = 10) were married (Table 1).

**Table 1:** Demographic characteristics of respondents (N = 28).

Participants	Age	Gender	Marital Status	Years of Experience
Midwife 1	25	Female	Single	3–5 year
Midwife 2	26	Female	Single	3–5 year
Midwife 3	25	Female	Single	3–5 year
Midwife 4	27	Female	Single	3–5 year
Midwife 5	25	Female	Single	3–5 year
Midwife 6	35	Female	Married	11 year and more
Midwife 7	25	Female	Single	3–5 year
Midwife 8	25	Female	Single	2 year and less
Midwife 9	34	Female	Single	6–10 year
Midwife 10	31	Female	Single	6–10 year
Nurse 1	28	Female	Married	3–5 year
Nurse 2	35	Female	Single	11 year and more
Nurse 3	51	Female	Married	11 year and more
Nurse 4	24	Female	Single	2 year and less
Nurse 5	24	Female	Single	2 year and less
Nurse 6	31	Male	Married	6–10 year
Nurse 7	23	Female	Single	2 year and less
Nurse 8	27	Female	Single	3–5 year
Nurse 9	26	Female	Single	3–5 year
Nurse 10	43	Female	Married	11 year and more
Nurse 11	35	Female	Married	11 year and more
Nurse 12	32	Female	Married	6–10 year
Doctor 1	32	Female	Single	6–10 year
Doctor 2	30	Female	Married	6–10 year
Doctor 3	30	Male	Single	6–10 year
Doctor 4	26	Female	Single	2 year and less
Doctor 5	36	Female	Married	11 year and more
Doctor 6	43	Male	Married	11 year and more

### 3.2 Themes

**Fig. 1:**
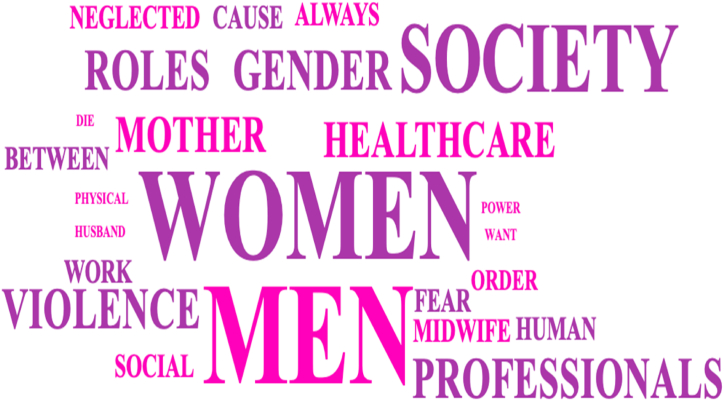
Distribution of the most frequently repeated words.

When the findings were examined after the individual interviews held about the views of healthcare professionals on gender roles, sub-codes and 6 main themes were determined (Table 2). These themes are “The neglect of women by society,” “Using force on women,” “Seeing women as sexual objects,” “Having a say in society,” “Glorified masculinity and femininity roles,” and “Gender equality.” (Fig. 1)

**Table 2:** Themes determined and classified from qualitative data.

Theme	Subtheme
Neglect of Women by Society	Being valueless Not being noticed Being despised Feeling helpless Being scorned
Using Force on Women	Pressure Fear Force Dominance
Seeing Women as Sexual Objects	Stigmatization Depreciation Judging
Glorified Masculinity and Femininity Roles	Sacred Continuity of the living race Building society Deification
Having a say in society	Being Listened to by Others Being respected Freedom Being valued
Gender Equality	Right Desexualization Competence

**Fig. 2:**
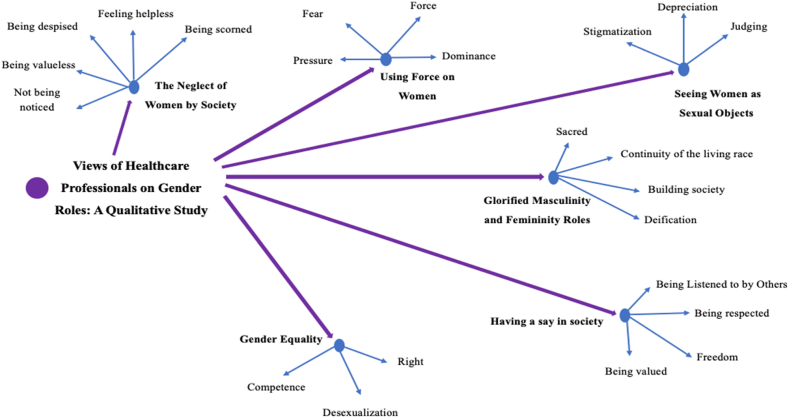
Theme-subcode hierarchy chart.

#### 3.2.1 Theme 1: the neglect of women by society

In their views on gender roles, healthcare professionals reported that women were not given importance due to being neglected, not being noticed, not being seen by society, and the responsibilities attributed to women by society, such as being only a mother.

At the beginning of human history, women had a very important place in society. As human history progressed and societies turned into complex structures, women were overshadowed by men. One of the healthcare professionals stated that women had been neglected throughout human history as follows: *"Since their creation, women have been oppressed, despised, and neglected individuals and not been so aware of their rights." (Midwife 1)*

The gender stereotypes assigned to professions by society have also caused a distinction between women's and men's work and the professions they can turn to, and this gender-based distinction has led to the perception of some professions as women's professions and some professions as men's professions over time. While women are usually associated with professions that include taking care of children, men are rather associated with professions that require muscle strength or professions such as management. To explain this situation, one of the healthcare professionals participating in the study said, *"Today, statements such as 'a woman cannot work like a man or a woman cannot do the work that a man does' cause women to be neglected. However, women can do many things that men can and can't do." (Midwife 3)*.

Healthcare professionals asserted that women were often associated with household chores, motherhood, and childcare in society. They defined the most sacred role that gender imposes on women as motherhood. *“When we look at our gender roles, I see that women are put under more workload, and women adhere to what needs to be done by society not only by being a mother and a woman." (Nurse 1) "A woman's status in society is a mother and a wife, but she is never considered a human being. The woman lives in society as a worthless being." (Midwife 4)*

Neglected by male-dominated value judgments in patriarchal societies, the woman has felt humiliated and helpless in all matters related to her femininity. *“Sometimes, when I ask a question to my female patients, their husbands say, 'she does not know, she cannot answer, I have to say' for some questions. In fact, they are questions related to the woman's life, her body. Men may even think women are incapable of answering some questions." (Midwife 5)*. In this statement, it is demonstrated that women's right to speak is prevented by men, even in matters related to their own bodies. Furthermore, it is seen that women have been deprived of rights such as education and inheritance since their childhood. Healthcare professionals said, *"We see that women do not have a say in many things for which women are seen worthless. We witness that girls are not counted as children, they are not educated, they are deprived of inheritance." (Doctor 2) and "There are still children/women in our country who are not sent to school, high school, university by their fathers as they are women. There are women who are neglected because they are mothers, and their only duty is to be a mother." (Nurse 2).*

#### 3.2.2 Theme 2: using force on women

This theme is related to practices such as pressure, force, authority, and dominance applied to women and the manifestation of fear in women due to these practices, according to the views of healthcare professionals on gender roles.

Healthcare professionals stated that psychological violence against women was frequently experienced in today's social order, insult was not prevented, and violence against women was normalized. Physical characteristics of men cause them to use pressure and force on women. *“We are living in a social order where women are oppressed and men are at the forefront. Women say, 'whatever my husband or brother says'." I see this fear even in patients. ‘Whatever my husband says, it should happen. I cannot express myself, my husband should'." Because there is an inequality of power between men and women - I'm talking about physical power - women have a fear of men. Murder of women is one of the biggest inequalities that men practice on women. I leave my boyfriend, and I die. I want to get divorced, and I die. I suffer violence, and I die. Is there anything beyond this?" (Midwife 1).* As a result of these inequalities of power between genders, women are scared. Men who are aware of the fear of women increase their pressure on women. This situation causes women to feel incompetent in their roles within the social order. Healthcare professionals expressed this situation in two ways:

“I think men cause a sense of incompetency as a result of their psychological pressure on women, which then leads to a vicious cycle resulting in women being less active in some areas of their lives." (Doctor 4)

*“Men want women to see them as strong and intelligent and to think that they need men."* (Midwife 2)

Another healthcare professional stated that men were always like vigilant weapons against women in all matters as follows:

“Men have always been like vigilant weapons against women because they see themselves stronger than women. There is only one norm in our society, and according to it, men use force on women, they impinge on women, they are stronger than women, and they can use both psychological violence and physical violence on them." (Midwife 3)

In patriarchal societies, women shape the stereotypes related to the social order in order to cope with patriarchy. In other words, women show the pressure applied by men in any period of their life cycles by establishing authority and control over other women. Thus, women play a key role in the reproduction of patriarchy itself. Looking at the statements of healthcare professionals about the reproduction of patriarchy, *"I think that men are more dominant as a return of a patriarchal society. This gender dominance also comes from the family, and this situation is changing culturally. For example, gender dominance is higher in Eastern culture than in Western culture. The woman suffers oppression sometimes by her mother-in-law and sometimes by her sister-in-law." (Midwife 4), "Men can be into showing power and try to oppress the other person. Women, on the other hand, may have difficulty controlling the change in their mood. Actually, women force women more." (Doctor 5)*

Healthcare professionals stated that, in societies where men were dominant, men were symbolized by their freedoms, whereas women's thoughts and behaviors about freedom were prevented by society. *“The male dominance of the part attributed to us … They are responsible for everything. Supposedly, men are above everything, his words need to be listened to in every way, and everything he does needs to be approved and accepted. There is an imposition that women should be under the domination of men." (Midwife 6)*

Healthcare professionals expressed that every woman today had the opportunity to choose whether or not to be a mother freely. The right to reproduce is one of the basic human rights, and every individual has the right to decide whether or not to have children. Pregnancy, which is a biological competence specific to women to ensure the continuity of the human race, is carried to the cultural area with patriarchal codes, transforming into a series of sanctions around the concept of "motherhood." Women are forced to become mothers by society, although they do not want to. *“Unfortunately, there are women who are forcibly deprived of the 'right to live,' although it is a fundamental right, and the law is not able to protect it. I believe that there are women who are forced to become mothers although they do not want to become." (Nurse 2)*

#### 3.2.3 Theme 3: seeing women as sexual objects

This theme is related to the fact that women are regarded as sexual objects in many areas according to the views of healthcare professionals on gender roles.

The image of women in today's society was stated by healthcare professionals as a subject associated with sexuality. A woman is seen as a sexual object rather than an individual and is valued for her sexual characteristics. *“Men use women as sexual objects in many places, especially in working life, for the purpose of attracting customers, and they continue to do so." (Midwife 1) "Since women are regarded as sexual objects deprived of every right and freedom in our society, women are given little importance in society. Men have no tolerance for women who have economic freedom and earn more than them since they see women only as sexual objects who give birth, take care of children, and cook." (Midwife 9) "Although women are strong, compassionate, and loving individuals, people in our society see women rather as sex* objects *or something like that." (Midwife 3)*

According to the statements of healthcare professionals, the word "woman" is divided into two as girl and woman in society, and although there is no biological gender definition as a girl, a woman who has not had sexual intercourse before is referred to as a "girl" and a woman who has had sexual intercourse is referred to as a "woman." *The worst part is that while the word 'woman' makes me feel strong, society attributes the word only to virginity. If you are a virgin, you are a girl; if you are not a virgin, you are a woman." (Midwife 4)*

Healthcare professionals expressed that, by looking at proverbs, individuals' positions and value judgments in the society they lived in could be reached. One of the healthcare professionals said, *"Gender differences bring about different physical and personal characteristics. The roles determined by these characteristics have changed culturally and traditionally and are degenerated in our society so severely that they are reflected in our proverbs." (Doctor 5)*

#### 3.2.4 Theme 4: glorified masculinity and femininity roles

This theme is related to how individuals are glorified by society according to the views of healthcare professionals on gender roles.

Healthcare professionals mentioned that power was symbolized by men in the patriarchal social structure. Although the word 'God' is mentioned as a supergender being, God is expressed as a man. The fact that someone creating the universe is strong comes to mind and power symbolizes men, the man is subconsciously glorified as a model of God. *“Men lead the patriarchal order that has been at the forefront from past to present. It is said that God does not have a gender, but some things such as our 'father' or 'ay dede' (moon) are deified as men." (Midwife 1)*

Healthcare professionals who took part in the study stated that the word 'woman' was an elite word and referred to a sacred power as follows: *"I think being a woman is very elite and very beautiful. I am so thankful that I am a woman. Motherhood is the crowned version of a woman. That is, the woman is a princess, and she becomes a queen when she becomes a mother. There is no higher level." (Midwife 6) "The word 'woman' means a great and sacred power to me." (Midwife 9)*

Moreover, according to healthcare professionals, the fertility of women is extremely important for the continuity of the living race. Healthcare professionals explained this situation as follows: *"In my opinion, women are the most valuable thing in this world, I do not say it because I am a woman myself, but a woman gives birth to a man. After all, where does our life start? It starts in the womb of a mother. Then, the first person we need after birth, or the one who benefits us the most, is the mother." (Midwife 6) "The woman is an individual who forms the first structure of a family. This is because motherhood is a special concept, and I think that the higher the place of women in society is, the better conditions our children and spouses will have." (Nurse 1) "I consider the woman the greatest element for the survival of the living race. I believe she is the greatest quality that should exist in child raising, working, and interpersonal communication." (Midwife 10)*

Healthcare professionals asserted that society was based on the family, and the family was based on the presence of women. Women, one of the basic building blocks of the family, play a significant role in the emergence and continuity of society. *“Women are the most fundamental and influential factor in the order and construction of society.” (Nurse 7)* Another healthcare professional said, *“Women are the most important part of society. There could not have been a society without women.” (Doctor 2)*

#### 3.2.5 Theme 5: having a say in society

This theme is related to the fact that women can have a say in society just like men can, they are respected and listened to, can express their thoughts and behave freely, and can assume their own responsibilities according to the views of healthcare professionals on gender roles.

While women and men learn different role patterns, they are influenced by the attitudes and behaviors of women and men in society. Hence, the social environment is the most important resource for learning to be a woman and a man. Nowadays, the social environment, which has changed with the development of societies, has also influenced the roles and responsibilities of women, causing an increase in employment in all areas of society. One of the healthcare professionals explained that the change in the social environment affected gender roles as follows: *"Gender roles have started to lose their influence today because women have become more educated, more knowledgeable, and are able to speak up. This has shown that women exist and everything should be common." (Midwife 8)* Furthermore, one of the healthcare professionals clearly expressed that the more a woman values herself, the more she will be valued as follows: *"You are valued in society as much as you feel valued. Whatever the status of a woman is in a family, that is her status in society." (Nurse 10)*

One of the healthcare professionals said, *"The place of women in working life continues to increase every day. Women's roles in the workplace are growing and evolving. Moreover, these roles and words of women have begun to be given importance by others. Socially, importance is attached to women's education and having a profession." (Midwife 5)*, underlining that the place of women in working life increases every day. Furthermore, one of the healthcare professionals explained the importance of women in working life and the effects of working life on women as follows: *"A woman's work is really very important for her to have a say somewhere, so I think the fact that she can work means people listen to and respect her in the society where she works. A working woman is always more respected in society." (Midwife 3)*

As the status of women in society increases, future generations will be affected positively. *“As long as we live in a world where a man is proven to be mentally more limited, the dominance of the role of women, the woman will have dominant and diverse roles, such as being a mother to the child, a guide to the husband, a psychologist to the husband or the child … In every way, women are highly valuable and versatile. Therefore, women should be extremely valued and respected in society." (Midwife 6)*

#### 3.2.6 Theme 6: gender equality

This theme is related to gender equality in society according to the views of healthcare professionals on gender roles.

Healthcare professionals stated that equality between men and women should be in all areas as follows: *"From a social perspective, I think that women should not sit at home or give birth and get stuck at home when men are working. They should be able to both start a family and work. I think men and women should have equal rights. A man should be able to take care of his children in the same way and should be able to work." (Midwife 2)*

Healthcare professionals explained the delivery of equal care while providing health services, without gender-based discrimination, as follows: *"I wish men and women could be valued in the same way both at work and at home or could cooperate." (Doctor 3) "Everyone should be approached with the same treatment regardless of gender in the health sector. I adopt this principle." (Nurse 4)*

Moreover, healthcare professionals suggested that the rights granted to men in working life should also be given to women, women should be seen as individuals, not as a gender, and they are competent enough to do the same jobs as men.

“Men and women are the genders with which people identify themselves. Neither of them has to have a separate or special place in society." (Doctor 4)

“In the role that exists in our society, women are considered naive and easy-going whereas men are seen as the dominant character. However, a common ground should be found." (Nurse 9)

*“There are two genders in society. Male and female, and both are equal."* (Midwife 5)

“I believe that all individuals should do everything, regardless of gender, to the extent of their abilities." (Doctor 2)

*“My opinion is that these roles should support each other. For example, the meticulous and considerate nature of women and the strength and protective nature of men should complete the relationships. Gender roles should not outweigh each other." (Doctor 5).* (Fig. 2)

## 4 Discussion

Healthcare professionals who took part in the study stated, on the one hand, that women were exposed to situations such as oppression, neglect, stigmatization, and exclusion by society. On the other hand, they stated that women were valued and given importance by society. Moreover, according to healthcare professionals, gender roles differ over time and positively affect the status of women in society. In line with these findings, the opinions of healthcare professionals about gender roles were discussed in light of the literature.

The biological dimension of gender and gender roles are different from each other. Gender roles refer to a process that starts from birth and is constructed by society [16]. There are two systems in these processes constructed by society. These two systems include men and women. While women are associated with descriptions such as weak, vulnerable, and over-emotional, men are associated with descriptions such as dominant, sexually aggressive, and physically strong [16,17]. The study by Edwards et al. (2021) reported that the power inequalities applied by men on women were accepted by societies, and violence was normalized. Although these power inequalities between the genders cause women to feel incompetent, healthcare professionals expressed that the power inequalities applied socially by men on women are structured and transferred intergenerationally [11,17,18].

According to gender roles, women are expected to be warm, polite, sensitive, caring for their children, and patient, whereas men are expected to have leadership skills, be determined, be logical, and have high self-confidence. Because of these roles, women are associated with their responsibilities in the family and at home, while men are associated with career and management [19,20]. According to healthcare professionals, women have been exposed to stereotypes such as “They cannot work like men” and “Women should only be mothers” since childhood. This situation causes women to be neglected in many areas.

Inequalities between women and men are also observed in occupational groups. Women and men are discriminated according to their gender in their professions, and only one-third of the professions are equally represented by men and women [21].

In the literature, it has been reported that women spend more than 100 min daily on household chores than men [21]. This situation results from the fact that the primary role of women is the maternal role. Society thinks that women's skills are limited only to family order and childcare [22].

It is known that a matriarchal order existed at the beginning of humanity. Mother-child bond forms the basic bond of culture, religion, and coexistence. Motherhood refers to the biological and psychological dimension of women and their superiority over men. Although women give life to a new being with their bodies and ensure social progress, women have been devalued by societies as societies have become complex structures [16,23]. Women and men have equal rights in all matters in today's democratic states of law, but gender roles prevent women from exercising these rights. This situation negatively influences women's health and well-being [11]. Healthcare professionals expressed that women are deprived of inheritance nowadays just because they are women, girls are not considered children and are not educated.

Healthcare professionals who took part in the study reported that women's right to speak was prevented by men, even in matters related to their own bodies. This situation prevents women's physical autonomy and restricts their freedom [9]. Considering the literature, the fact that healthcare professionals provide services based on gender equality and health equality will enable women to have a say on their bodies [9,11,24]. Furthermore, healthcare professionals emphasized the importance of providing the same care to everyone in the health sector, regardless of gender inequality [25].

The place of women in society has begun to differ over time. Nowadays, women have become liberated, and discrimination against women has decreased [26]. To ensure gender equality and change the roles assigned to women in society, societies should eliminate the obstacles in front of women and see them as equal individuals, enabling them to become a part of society [9]. The healthcare professionals participating in the study asserted that the roles of women in society were increasing every day, and they were starting to have a say in society. Additionally, they underlined that society paid attention to women's education and having a profession.

Gender equality is one of the most important determinants of health because gender roles are effective in the health risk behaviors of individuals [11,27]. In the definitions of healthcare professionals for gender equality, women and men should be equal, regardless of gender.

In gender roles, women and men should have the same jobs, regardless of gender, have equal rights, and share chores at home. Gender roles should not prevail over each other. In contrast, they should complement each other [4,28].

## 5 Study limitations

Since this research was conducted with healthcare professionals working in an institution, the sample represents only this institution. Therefore, the research results cannot be generalized, which is one of the study's limitations.

## 6 Conclusion

In society, women are exposed to inequalities since they are women. The woman assumes the role of a mother dealing with household chores or caring for children, whereas the man assumes the role of a worker, earner, and manager. This situation is associated with the fact that women experience negativities due to gender roles and gender inequalities in many areas, especially in the field of health. Healthcare professionals should treat everyone equally to improve the quality of care they provide while delivering health services. Care to be provided equally by healthcare professionals will ensure that individuals are protected from possible conditions such as illnesses or disabilities, especially by positively affecting women's lives.

## Author contribution statement

Zeynep Dilşah KARAÇAM YILMAZ: Conceived and designed the experiments; Performed the experiments; Analyzed and interpreted the data; Contributed reagents, materials, analysis tools or data; Wrote the paper.

Tülay YILMAZ: Conceived and designed the experiments; Performed the experiments; Analyzed and interpreted the data; Wrote the paper.

Eda TOKMAN: Performed the experiments; Contributed reagents, materials, analysis tools or data; Wrote the paper.

## Data availability statement

The data that has been used is confidential.

## Additional information

No additional information is available for this paper.

## Author agreement

We confirm that this work is original and has not been published elsewhere, nor is it currently under consideration for publication elsewhere.

## Declaration of competing interest

The authors declare that they have no known competing financial interests or personal relationships that could have appeared to influence the work reported in this paper.
